# Supplementary material for: COVID-19 in Pakistan: A national analysis of five pandemic waves
Source: PLoS One. 2023 Dec 29;18(12):e0281326. doi: 10.1371/journal.pone.0281326 (PMC10756537; doi:10.1371/journal.pone.0281326)
Supplement: S1 Table — (DOCX) [file pone.0281326.s001.docx]

**Table S1. Calculated ratio variables and their descriptions**

| Variable Name | Description |
| --- | --- |
| Test-to-Case Ratio | The ratio of tests performed for each confirmed COVID-19 case |
| COVID-19 Positivity | Daily new cases as a percentage of daily new tests |
| Oxygen-Admitted Ratio | The number of COVID-19 patients on oxygen beds as a proportion of the total COVID-19 hospital admissions in Pakistan |
| Oxygen Utilization Ratio | The number of people on oxygen beds as a proportion of the total oxygen beds allocated for COVID-19 in Pakistan |
| Ventilators Utilization Ratio | The number of people on ventilators as a proportion of the total ventilators allocated for COVID-19 in Pakistan |
| Length of Hospital Stay | The ratio of the total number of patients who are in the hospital at a given moment to the total number of daily new patients who are admitted to the hospital. |
