# Supplementary material for: COVID-19 in Pakistan: A national analysis of five pandemic waves
Source: PLoS One. 2023 Dec 29;18(12):e0281326. doi: 10.1371/journal.pone.0281326 (PMC10756537; doi:10.1371/journal.pone.0281326)
Supplement: Attachment — Submitted filename: PONE-D-23-01793_reviewer.pdf [file pone.0281326.s002.pdf]

PLOS ONE

COVID-19 in Pakistan: A national analysis of five pandemic waves

--Manuscript Draft--

**Manuscript Number:**	PONE-D-23-01793
**Article Type:**	Research Article
**Full Title:**	COVID-19 in Pakistan: A national analysis of five pandemic waves
**Short Title:**	COVID-19 waves in Pakistan
**Corresponding Author:**	Adnan Ahmad Khan<br>Research and Development Solutions<br>Islamabad, ICT PAKISTAN
**Keywords:**	COVID-19 waves; Variants of concern; Infectious diseases; Daily COVID-19 cases; Daily COVID-19 deaths
**Abstract:**	Objectives<br>The COVID-19 pandemic showed distinct waves where cases ebbed and flowed. While each country had slight, nuanced differences, lessons from each wave with country-specific details provides important lessons for prevention, understanding medical outcomes and the role of vaccines. This paper compares key characteristics from the five different COVID-19 waves in Pakistan.<br>Methods<br>We used specific criteria to define COVID-19 waves, and key variables such as COVID-19 tests, cases, and deaths with their rates of change to the peak and then to the trough were used to draw descriptive comparisons. Additionally, a linear regression model estimated daily new COVID-19 deaths in Pakistan.<br>Results<br>Pakistan saw five distinct waves, each of which displayed the typical topology of a complete infectious disease epidemic. The time from wave-start to peak became progressively shorter, and from wave-peak to trough, progressively longer. Each wave appears to also be getting shorter, except for wave 4, which lasted longer than wave 3. A one percent increase in vaccinations increased daily new COVID-19 deaths by 0.10% (95% CI: 0.01, 0.20) in wave 4 and decreased deaths by 0.38% (95% CI: -0.67, -0.08) in wave 5.<br>Conclusion<br>Each wave displayed distinct characteristics that must be interpreted in the context of the level of response and the variant driving the epidemic. Key indicators suggest that COVID-19 preventive measures kept pace with the disease. Waves 1 and 2 were mainly about prevention and learning how to clinically manage patients. Vaccination started late during Wave 3 and its impact became apparent on hospitalizations and deaths in Wave 5. The impact of highly virulent strains Alpha/B1.1.7 and Delta/B.1.617.2 variants during Wave 3 and milder but more infectious Omicron/BA.5.2.1.7 are apparent.
**Order of Authors:**	Taimoor Ahmad
	Mujahid Abdullah
	Abdul Mueed
	Faisal Sultan
	Ayesha Khan
	Adnan Ahmad Khan
**Additional Information:**	
**Question**	**Response**
**Financial Disclosure**<br><br>Enter a financial disclosure statement that describes the sources of funding for the	This work was supported, in whole or in part, by the Bill & Melinda Gates Foundation [grant number: INV-025171]. Under the grant conditions of the Foundation, a Creative Commons Attribution 4.0 Generic License has already been assigned to the Author Accepted Manuscript version that might arise from this submission. The funders had no

Powered by Editorial Manager® and ProduXion Manager® from Aries Systems Corporation

work included in this submission. Review the [submission guidelines] for detailed requirements. View published research articles from *PLOS ONE* for specific examples.<br><br>This statement is required for submission and **will appear in the published article** if the submission is accepted. Please make sure it is accurate.<br><br>**Unfunded studies**<br>Enter: *The author(s) received no specific funding for this work.*<br><br>**Funded studies**<br>Enter a statement with the following details:<br>• Initials of the authors who received each award<br>• Grant numbers awarded to each author<br>• The full name of each funder<br>• URL of each funder website<br>• Did the sponsors or funders play any role in the study design, data collection and analysis, decision to publish, or preparation of the manuscript?<br>• **NO** - Include this sentence at the end of your statement: *The funders had no role in study design, data collection and analysis, decision to publish, or preparation of the manuscript.*<br>• **YES** - Specify the role(s) played.<br><br>* typeset	role in study design, data collection and analysis, decision to publish, or preparation of the manuscript.
**Competing Interests**<br><br>Use the instructions below to enter a competing interest statement for this submission. On behalf of all authors, disclose any competing interests that could be perceived to bias this work—acknowledging all financial support and any other relevant financial or non-financial competing interests.<br><br>This statement is required for submission and **will appear in the published article** if the submission is accepted. Please make sure it is accurate and that any funding sources listed in your Funding Information	The authors have declared that no competing interests exist.

Powered by Editorial Manager® and ProduXion Manager® from Aries Systems Corporation

later in the submission form are also declared in your Financial Disclosure statement.<br><br>View published research articles from *PLOS ONE* for specific examples.<br><br>**NO authors have competing interests**<br><br>Enter: *The authors have declared that no competing interests exist.*<br><br>**Authors with competing interests**<br><br>Enter competing interest details beginning with this statement:<br><br>*I have read the journal's policy and the authors of this manuscript have the following competing interests: [insert competing interests here]*<br><br>* typeset	
**Ethics Statement**<br><br>Enter an ethics statement for this submission. This statement is required if the study involved:<br><br>• Human participants<br>• Human specimens or tissue<br>• Vertebrate animals or cephalopods<br>• Vertebrate embryos or tissues<br>• Field research<br><br>Write "N/A" if the submission does not require an ethics statement.<br><br>General guidance is provided below. Consult the submission guidelines for detailed instructions. **Make sure that all information entered here is included in the Methods section of the manuscript.**	N/A

**Format for specific study types**<br><br>**Human Subject Research (involving human participants and/or tissue)**<br>• Give the name of the institutional review board or ethics committee that approved the study<br>• Include the approval number and/or a statement indicating approval of this research<br>• Indicate the form of consent obtained (written/oral) or the reason that consent was not obtained (e.g. the data were analyzed anonymously)<br><br>**Animal Research (involving vertebrate animals, embryos or tissues)**<br>• Provide the name of the Institutional Animal Care and Use Committee (IACUC) or other relevant ethics board that reviewed the study protocol, and indicate whether they approved this research or granted a formal waiver of ethical approval<br>• Include an approval number if one was obtained<br>• If the study involved *non-human primates*, add *additional details* about animal welfare and steps taken to ameliorate suffering<br>• If anesthesia, euthanasia, or any kind of animal sacrifice is part of the study, include briefly which substances and/or methods were applied<br><br>**Field Research**<br><br>Include the following details if this study involves the collection of plant, animal, or other materials from a natural setting:<br>• Field permit number<br>• Name of the institution or relevant body that granted permission	
**Data Availability**<br><br>Authors are required to make all data underlying the findings described fully available, without restriction, and from the time of publication. PLOS allows rare exceptions to address legal and ethical concerns. See the PLOS Data Policy and FAQ for detailed information.	No - some restrictions will apply

A Data Availability Statement describing where the data can be found is required at submission. Your answers to this question constitute the Data Availability Statement and **will be published in the article**, if accepted.<br><br>**Important:** Stating 'data available on request from the author' is not sufficient. If your data are only available upon request, select 'No' for the first question and explain your exceptional situation in the text box.<br><br>Do the authors confirm that all data underlying the findings described in their manuscript are fully available without restriction?	
**Describe where the data may be found in full sentences. If you are copying our sample text, replace any instances of XXX with the appropriate details.**<br><br>• If the data are **held or will be held in a public repository**, include URLs, accession numbers or DOIs. If this information will only be available after acceptance, indicate this by ticking the box below. For example: *All XXX files are available from the XXX database (accession number(s) XXX, XXX.).*<br>• If the data are all contained **within the manuscript and/or Supporting Information files**, enter the following: *All relevant data are within the manuscript and its Supporting Information files.*<br>• If neither of these applies but you are able to provide **details of access elsewhere**, with or without limitations, please do so. For example:<br><br>*Data cannot be shared publicly because of [XXX]. Data are available from the XXX Institutional Data Access / Ethics Committee (contact via XXX) for researchers who meet the criteria for access to confidential data.*<br><br>*The data underlying the results presented in the study are available from (include the name of the third party*	The data was provided to the Akhter Hameed Khan Foundation (AHK-F) team for this study as part of its work with Pakistan's Federal Ministry of National Health Services, Regulations & Coordination (MoNHSR&C) and the National Command & Operation Centre (NCOC) in Islamabad, which are leading Pakistan's response to the COVID-19 pandemic. The AHK-F team has provided analytical support to the above entities, and such created knowledge that has directly informed pandemic policy-making in Pakistan. COVID-19 data is compiled and shared in daily National Situation Reports, or Sitreps, by the National Emergency Operation Centre (NEOC). Each day's Sitrep is compiled as a PDF file. The data used for this study was manually compiled from these PDF files and then used in STATA. The parentage of this data is with the NCOC and the MoNHSR&C. The AHK-F team received this data with the express understanding that it would be kept confidential. However, the data can be obtained independently from the NEOC, through a data request procedure, which is subject to approval from the MoNHSR&C. The data request itself is to be addressed to: Dr. Shahzad Baig, National Coordinator, National Emergency & Operation Center, D Block, EPI Building, Chak Shahzad, Park Road, Islamabad. Email: eocpakistan@gmail.com Phone: +92-51-8730879.<br>The data on Oxford Health and Containment Index is taken from and publicly available at the following GitHub repository: https://github.com/OxCGRT/covid-policy-tracker/tree/master/data<br><br>Ethical Consideration and data privacy:<br>AHK-F team was working as consultant agency and was providing analytical reports. As per statement data was taken from AHK foundation. Did any permission was sought from NCOC and Ministry of Health as they were guardian.

and contact information or URL).<br>• This text is appropriate if the data are owned by a third party and authors do not have permission to share the data.<br><br>* typeset	
Additional data availability information:	

Powered by Editorial Manager® and ProduXion Manager® from Aries Systems Corporation

Manuscript
Click here to access/download;Manuscript;Wave_analysis_Manuscript.docx

COVID-19 in Pakistan: A national analysis of five pandemic waves

Short title: COVID-19 waves in Pakistan

Taimoor Ahmad[1], Mujahid Abdullah[1], Abdul Mueed[1], Faisal Sultan[2,3], Ayesha Khan[1], Adnan Ahmad Khan[2,4*]

[1]Akhter Hameed Khan Foundation, Islamabad, Pakistan

[2]Ministry of National Health Services, Regulation and Coordination, Islamabad, Pakistan

[3]Shaukat Khanum Memorial Cancer Hospital & Research Centre, Lahore, Pakistan

[4]Research and Development Solutions, Islamabad, Pakistan

***Corresponding Author**

Email: adnan@resdev.org

Disclosures:

This work was supported, in whole or in part, by the Bill & Melinda Gates Foundation [grant number: INV-025171]. Under the grant conditions of the Foundation, a Creative Commons Attribution 4.0 Generic License has already been assigned to the Author Accepted Manuscript version that might arise from this submission.

ABSTRACT

Objectives

The COVID-19 pandemic showed distinct waves where cases ebbed and flowed. While each country had slight, nuanced differences, lessons from each wave with country-specific details provides important lessons for prevention, understanding medical outcomes and the role of vaccines. This paper compares key characteristics from the five different COVID-19 waves in Pakistan.

Methods

describe little bit about specific criteria

We used specific criteria to define COVID-19 waves, and key variables such as COVID-19 tests, cases, and deaths with their rates of change to the peak and then to the trough were used to draw descriptive comparisons. Additionally, a linear regression model estimated daily new COVID-19 deaths in Pakistan.

Results

Pakistan saw five distinct waves, each of which displayed the typical topology of a complete infectious disease epidemic. The time from wave-start to peak became progressively shorter, and from wave-peak to trough, progressively longer. Each wave appears to also be getting shorter, except for wave 4, which lasted longer than wave 3. A one percent increase in vaccinations increased daily new COVID-19 deaths by 0.10% (95% CI: 0.01, 0.20) in wave 4 and decreased deaths by 0.38% (95% CI: -0.67, -0.08) in wave 5.

Conclusion

Each wave displayed distinct characteristics that must be interpreted in the context of the level of response and the variant driving the epidemic. Key indicators suggest that COVID-19 preventive measures kept pace with the disease. Waves 1 and 2 were mainly about prevention and learning how to clinically manage patients. Vaccination started late during Wave 3 and its impact became apparent on hospitalizations and deaths in Wave 5. The impact of highly virulent strains Alpha/B1.1.7 and Delta/B.1.617.2 variants during Wave 3 and milder but more infectious Omicron/BA.5.2.1.7 are apparent.

INTRODUCTION

In Pakistan, the first case of COVID-19, a novel and little-understood disease, was detected on February 26, 2020. Being a developing country with limited resources, crumbling health infrastructure and low health expenditure (1), Pakistan has no past experience with pandemics and a high burden of communicable diseases (2). As of February 23, 2022, the country had fully vaccinated 43% of its total population (3) and the Omicron variant of COVID-19 was the dominant strain (4). These factors make Pakistan a high-risk country, with a large pool of infection-susceptible people.

The emergence of COVID-19 has arguably been the biggest social and economic disruption in Pakistan in recent history. The pandemic has largely manifested itself in five distinct waves each of which have a rise, plateau and trough in cases, followed by a period of dormancy, after which the incidence of COVID-19 infections begins to rise again. Thus, each individual wave follows a four stage pattern followed by endemicity that has been seen for many infectious disease epidemics (12). Beyond anecdotal observation, there is evidence that this is happening with COVID-19 as well (11). What sets COVID-

19 apart is that after completion of an individual wave, a new one would come along shortly, rather than much long, for example, annual, recurrences for influenza etc. This pattern has been seen all across the globe (5–10) with the timing of COVID-19 waves in different countries broadly coinciding (11).

In this context, current literature on COVID-19 largely focuses on high-income countries during the initial waves (2,13–17), or aggregated at a regional level (5,6). Given the different capacities of countries to manage the pandemic (17), there is a need to explore the characteristics of the subsequent pandemic waves in a developing country context, preferably with granularity of a country-level analysis (2,13–17).

To this end, this paper explores how each of the five waves of the COVID-19 pandemic in Pakistan differed from each other in terms of the total duration of each wave, the time from the start of each wave to its peak, and from its peak to the wave's trough; the length of time in between each wave; as well as key descriptive features like the total number of COVID-19 tests, COVID-19 cases, hospitalizations, deaths from COVID-19, and COVID-19 vaccinations in each wave. Additionally, this study statistically models the key factors that lead to deaths from COVID-19.

The rationale and justification for this article must be incoporated

There must be provincial and regional comparison

METHODS

Study design not clearly mentioned.

Criteria for COVID-19 waves

There were COVID-19 dashboard in each province. Why data from these dashboards were not reflected in study. These dashboards have all demographic and risk factors data.

We begin by retrospectively defining various time periods between 2020 and 2022 as distinct waves, based on existing literature (18). There are a total of 628 observations (daily set of indicators) across these five waves. Based on our criteria, the starting point of each COVID-19 wave is defined as the day with the lowest number of daily new COVID-

Provincial specific cultural and social, religious, education status, social mobalization, public compliance, administrative, infrastructural (etc) factors must be considered for NPI. Did these factors may have played a role as confounder.

19 cases preceding a consistent rise in these cases, before the peak of the respective COVID-19 wave. The end of each wave is defined as the day with lowest number of daily new COVID-19 cases following a 7-day decline; this number also needed to be lower than the cases on any of the 7 days that followed it (Table 1).

Data and variables

In order to estimate the pattern for COVID-19 throughout the five waves in Pakistan, we have used time series of various daily indicators from April 3, 2020 to February 23, 2022, which categorized into following themes:

i) Wave timespan
ii) COVID-19 tests
iii) COVID-19 cases
iv) Test-to-case ratio
v) COVID-19 positivity
vi) Hospitalization, treatment, and containment
vii) COVID-19 deaths Confounding factors for deaths like comorbidity may be considered
viii) COVID-19 vaccination What about fake vaccination scenario
ix) Policy environment

Several variables in the list above were transformed into ratios for the purpose of describing all five COVID-19 waves in Pakistan (Supplementary Table S1).

The data for all but two of the above variables, second doses of COVID-19 vaccines administered and the Oxford Containment and Health Index for COVID-19, was compiled from daily national situation reports (Sitreps). These Sitreps are prepared by the National

Emergency Operations Centre (NEOC) in Islamabad, Pakistan. Data in these Sitreps have served as the basis for all major COVID-19 policy decisions in Pakistan.

The data for second doses of COVID-19 vaccines administered was sourced directly from the National Command & Operation Centre (NCOC), Islamabad, Pakistan, which is the government forum that brings together the ministries of Health and Planning along with the military to determine pandemic policy and to coordinate the response. Data for the Oxford Containment and Health Index is taken from a publicly available dataset from the University of Oxford's Blavatnik School of Governance (19). This dataset is compiled by using qualitative information about the non-pharmaceutical interventions (NPIs) in a country and quantifying them into an index. A detailed methodology of the index calculation can be found in a working paper by the Blavatnik school (20).

Model Specification

For our model for COVID-19 deaths, we used a linear ordinary least square (OLS) regression carried out using STATA 17. The data as well as the model was divided into five distinct periods, representing the five waves of COVID-19 in Pakistan, as of February 2022. Regression analysis was performed separately on each wave and results were compared with each other to make logical inferences.

Our dependent variable is the Daily New COVID-19 Deaths in Pakistan in logarithmic form ($\mathbf{LnY_t}$); ϵ_t represents the error term capturing the effect of omitted variables. The specification of our linear OLS regression model is as following:

$$\boldsymbol{Ln\, Y_t = \beta_0 + \beta_1\, Ln\, X_{1t} + \beta_2\, Ln\, X_{2t} + \beta_3\, X_{3t} + \beta_4\, X_{4t} + \beta_5\, X_{5t} + \beta_6\, X_{6t} + \beta_7\, Ln\, X_{7t} + \epsilon_t}$$

We have chosen seven independent variables:

i) Log of daily new COVID-19 cases with 21-day delay ($\mathbf{LnX_{1t}}$);

ii) Log of daily new COVID-19 tests with 28-day delay ($\mathbf{LnX_{2t}}$);

iii) The Oxford Containment and Health Index for COVID-19 with 14-day delay ($\mathbf{X_{3t}}$);

iv) Time ($\mathbf{X_{4t}}$);

v) The number of people on ventilators as a proportion of the total admitted ($\mathbf{X_{5t}}$);

vi) The number of people on oxygen as a portion of the total admitted ($\mathbf{X_{6t}}$);

vii) Log of second doses of COVID-19 vaccines administered with 14- day delay ($\mathbf{LnX_{7t}}$)

Daily new COVID-19 cases were regressed with a 21-day lag, since among those who die from COVID-19 infection, death occurs between a median of 14 days (21) and 25 days (average of three weeks) after presenting symptoms (22,23). This is pertinent in the case of Pakistan, as most of the COVID-19 testing in the country has been symptomatic, i.e., done when someone develops symptoms of COVID-19 and hence either voluntarily gets tested or is prescribed by a medical professional to do so.

What about TTQ and Surveillance, close contacts. There is alot of TTQ Data. Consider this point

Given the delay for daily new cases, daily new COVID-19 tests were regressed with a delay of 28 days. This delay allows for the time it takes for someone to test positive for COVID-19 and for their symptoms to worsen (for example, by escalating to hospitalization, which takes nearly a week (24) before resulting in death. For vaccination, a 14-day lag was taken, as immunity from vaccines is generally understood to develop two weeks or longer after receiving a shot (25–27).

The Oxford Containment and Health Index is calculated out of 100 which means strict restrictions and a lower value indicate a smaller number of restrictions imposed on the

general population. This variable was regressed with a 14-day lag, as we assume that any new government restrictions will take approximately that long to have any effect. Additionally, the time variable is meant to capture any unmeasured or seasonal effects on COVID-19 deaths in Pakistan, such as an overall rate of increase/ decrease of daily deaths in each wave. We assume the error term is not correlated with any of the independent variable.

Can you explore more about. Assuming must be replaced with scientific prove and referencing

Diagnostic tests were performed on the estimated model to ensure the precision of the estimated coefficient: for heteroskedasticity, the Breusch-Pagan test was applied, whereas for serial correlation, the Durbin-Watson test was used. Variance inflation factor (VIF) was calculated for multicollinearity. Dickey-Fuller tests were run for each independent variable in our regression model. All of the variables were found to be stationary, fulfilling an important pre-requisite for our analysis (Table 1).

Table 1. Augmented Dicky-Fuller tests for unit roots

Variable	Test Statistics	P-Value	H_0: Random Walk
**Daily New COVID-19 Deaths**	-6.96	0.000	Stationary
**Daily New COVID-19 Cases**	-3.23	0.018	Stationary
**Daily New COVID-19 Tests**	-3.94	0.0017	Stationary
**Oxford Containment and Health Index**	-3.30	0.015	Stationary
**Ventilator-admitted Ratio**	-5.69	0.000	Stationary
**Oxygen-Admitted Ratio**	-3.00	0.034	Stationary
**Fully Vaccinated People**	-2.77	0.003	Stationary

RESULTS

Summary statistics

Pakistan experienced five distinct waves from 3rd April 2020 till 23rd February 2022 (Fig 1). The first wave lasted the longest (150 days), while the fifth one was the shortest (83

days). The fourth wave was remarkable for its relatively rapid upslope and a long tail, while the fifth wave showed the reverse pattern. The duration of each wave of COVID-19 in Pakistan was shorter than the preceding one apart from wave 4. After wave 1, each wave took less time to reach its peak and took longer to reach its trough apart from wave 5.

Figure 1. COVID-19 waves and daily new cases

The capacity to conduct tests expanded over time from an average of 17,142 tests daily during the first wave to 49,650 during fifth wave. The increase in the daily tests peaked during the fourth wave. The highest average daily number of cases (3147) were observed during the wave 3. The rate of increase of COVID-19 cases was highest during wave 4, but the rate of decline in cases after the peak of a wave was fastest during wave 5. Test to case ratio kept on increasing from 15 during the first wave to 57 during the fifth one. While total positivity varied across waves, the rate in daily change of positivity remained relatively unchanged apart from wave 2 and 3, where it is lower as compared to other waves (Table 2).

Table 2. Descriptive characteristics of each COVID-19 wave

Variables	Wave 1	Wave 2	Wave 3	Wave 4	Wave 5
**Dates**	April 3, 2020 to August 31, 2020	October 12, 2020 to February 16, 2021	February 23, 2021 to June 22, 2021	July 6, 2021 to November 29, 2021	December 7, 2021 to February 23, 2022
**Main Variants of Concern (VOCs)**	B.1	B.1.36	Alpha/ B1.1.7/ Delta/B.1.617.2	Delta/B.1.617.2	Omicron/BA.5.2.1.7
**Timespan**					
Duration of wave (days)	150	128	120	147	83
From wave Start till Peak (days)	72	57	55	31	52
From wave Peak till Trough (days)	78	71	65	116	31
**COVID-19 Tests**					

Total tests in the entire wave		2,571,244	4,638,219	5,458,041	7,148,130	4,120,909
Avg. Daily New COVID-19 Tests		17,142 (7,931)	36,236 (5,363)	45,484 (8,623)	48,627 (8341)	49,650 (7494)
Rate of Change of Daily New Tests	Up to Peak	380.9	216.5	701.1	836.6	540.4
Rate of Change of Daily New Tests	After Peak	-147.8	-101.0	-504.9	-286.4	-1137.6
**COVID-19 Cases**						
Total cases in the entire wave		293,752	246,188	377,668	320,333	221,825
Avg. Daily New COVID-19 Cases		1,958 (1,687)	1,923 (815)	3,147 (1,547)	2,179 (1548)	2,673 (2519)
Rate of Change of Daily New Cases	Up to Peak	93.9	60.9	94.0	161.0	155.9
Rate of Change of Daily New Cases	After Peak	-84.8	-40.0	-84.1	-48.0	-236.4
**Test-to-Case Ratio**						
Average Test-to-Case Ratio		15 (15)	23 (11)	19 (11)	44 (38)	57 (56)
Rate of Change of Test-to-Case Ratio	Up to Peak	4.1	-1.1	-0.4	-1.13	-3.31
Rate of Change of Test-to-Case Ratio	After Peak	84.5	0.3	0.7	1.36	0.99
**COVID-19 Positivity**						
Avg. Daily Positivity Ratio during the wave		11.3% (6.4%)	5.2% (1.8%)	6.8% (2.9%)	4.2% (2.6%)	4.9% (4.1%)
Rate of Change of Positivity Ratio	Up to Peak	0.23	0.15	0.10	0.23	0.22
Rate of Change of Positivity Ratio	After Peak	-0.28	-0.10	-0.11	-0.07	-0.30
**Hospitalization, Treatment, and Containment**						
Avg. COVID-19 Hospital Admissions at any given time		3,817 (2,479)	2,187 (818)	3,970 (1,366)	3,396 (1,776)	1125 (423)
Avg. Length of Hospital Stay (days)[a]		-	13	11	9	5
Avg. Daily New Hospitalization to Daily New Case Ratio (%)		-	8.8 (2.4)	12.4 (3.5)	22.7 (10.7)	15.7 (10.8)
Avg. Daily Recoveries and Hospital Discharges		1,870 (2,587)	1,746 (1,608)	2,990 (1,454)	2,272 (2,329)	2371 (3797)
Rate of Change of Daily Recoveries and Hospital Discharges (non-cumulative)	Up to Peak	23.4	14.3	69.9	202.4	28.76

Rate of Change of Daily Recoveries and Hospital Discharges (non-cumulative)	After Peak	-19.8	-6.6	-51.1	-57.7	-0.97
Avg. Stable-Admitted Ratio (%)		67% (27%)	20% (6%)	14% (5%)	7% (4%)	9% (4%)
Avg. Patients on Oxygen-Admitted Ratio (%)		27% (23%)	68% (5%)	75% (5%)	83% (2%)	81% (3%)
Avg. Number of Oxygen Beds in Use at any given time		865 (828)	1,521 (608)	2,995 (1,080)	2.803 (1,470)	918 (361)
Avg. Oxygen Beds Utilization Ratio (%)		18% (11%)	15% (6%)	24% (8%)	20% (10%)	7% (3%)
Avg. Number of Ventilators in Use at any given time		214 (163)	249 (90)	429 (145)	336 (153)	109 (36)
Avg. Ventilator-Admission Ratio (%)		7% (4%)	11% (1%)	11% (0.6%)	10% (2%)	10% (1%)
Avg. Ventilators Utilization Ratio (%)		17% (10%)	13% (5%)	20% (7%)	15% (7%)	5% (2%)
**COVID-19 Deaths**						
Total COVID-19 deaths in the entire wave		6,204	5,537	9,423	6,287	1,401
Avg. Daily New COVID-19 Deaths		41.4 (35.3)	43.3 (23.0)	78.5 (37.4)	42.8 (30.6)	16.9 (14.3)
Rate of Change of Daily New COVID-19 Deaths	Up to Peak	1.1	0.5	2.0	1.17	0.45
Rate of Change of Daily New COVID-19 Deaths	After Peak	-1.0	0.1	-1.9	-0.44	-0.81
Avg. Daily New COVID-19 Deaths - Daily Hospitalization Ratio (%)		11% (5%)	27% (13%)	23% (8%)	10% (4%)	7% (4%)
Avg. Daily New COVID-19 Deaths – Ventilator in use Ratio (%)		23% (17%)	17% (6%)	18% (5%)	12% (5%)	13% (8%)
Avg. Death-to-Case Ratio (%)		2.2% (0.8%)	2.3% (1.1%)	2.8% (1.2%)	2.2% (0.8%)	1.1% (1.0%)
**COVID-19 Vaccination**[b]						
Total Administration of 2nd doses of Vaccine		-	-	3,136,386	45,294,948	45,667,572
Avg. Daily New Administration of 2nd doses of Vaccine		-	-	26,356 (25,980)	308,129 (172,896)	550,212 (186,287)
**Policy Environment**						
Oxford Containment and Health Index		58.8 (7.6)	62.4 (4.0)	65.7 (6.3)	58.1 (9.4)	58.6 (5.5)

Standard deviations are reported in parentheses.

[a]Data for daily new COVID-19 hospitalizations was not available during wave 1.
[b]COVID-19 vaccines were not being administered during waves 1 and 2.

Hospitalizations were the highest for the first and the third wave and the least for the fifth, whereas duration of hospitalization fell linearly from an initial 13 days during the second wave to 5 days during the fifth. Hospitalizations became more specific over time in that, nearly two third of admitted patients during the first wave were stable, compared to 9% during the fifth one. The average stable-admitted ratio decreased continuously from wave 1 to wave 4 but increased slightly in wave 5. The rate at which people recovered from COVID-19 and/or were discharged from hospital was highest in wave 4 and slowest in wave 2.

The average Oxygen Beds-Admitted Ratio continuously increasing in each wave, reaching its maximum value in wave 4. During the first wave 27% of all admissions required oxygen, 7% needed a ventilator, compared to 81% and 10% respectively during the fifth wave. The average oxygen bed utilization followed a declining trend except for the third wave (24%) and was lowest in the fifth wave (7%). The average Ventilators Utilization Ratio shows that all available ventilators were not fully utilized in any of the five waves. The highest ratio was in wave 3 (20%) and the lowest ratio was in wave 5 (5%). These two ratios suggest that most critical patients were put on oxygen for recovery and a small proportion of these people were transferred to ventilators.

Deaths from COVID-19 were the highest during the third wave at 9,423, which also saw the highest daily number of deaths (78.5) and the highest rate of increase in daily deaths. Average daily deaths to hospitalization peaked during the second wave, while deaths to ventilator use was the highest during the first. Average death to case ratio was the highest

for the third wave but was in the 2.2-2.8% range, with the exception of the fifth wave when it was 1.1%.

Pakistan's vaccination drive started towards the end of wave 2, but full vaccination (i.e., people receiving both their doses) did not occur until the beginning of wave 3. Consequently, total and daily new second dose of vaccine administered was highest in wave 5. Government restrictions, measured by the Oxford Health and Containment Index, appear to have been comparable in each wave.

Regression results

The results indicate the daily new COVID-19 cases were a statistically significant determinant for daily new deaths in all five waves at 95% CI; a one-percentage increase in COVID-19 cases caused a 0.46- 0.69 % increase in deaths across the five waves (Table 3).

Table 3. Ordinary least squares (OLS) regression results for COVID-19 deaths

**LOG (Daily New COVID-19 Deaths)**	(1) Wave 1	(2) Wave 2	(3) Wave 3	(4) Wave 4	(5) Wave 5
**LOG (Daily New COVID-19 Cases) – 21-day delay**	0.455* [0.289,0.620]	0.685* [0.430,0.939]	0.691* [0.358,1.025]	0.677* [0.542,0.812]	0.506* [0.189,0.823]
**LOG (Daily New COVID-19 Tests) – 28-day delay**	0.648* [0.260,1.037]	0.308 [-0.179,0.795]	-0.162 [-0.645,0.322]	0.268 [-0.122,0.657]	0.296 [-0.771,1.364]
**Oxford Containment and Health Index – 14-day delay**	-0.027* [-0.050, -0.005]	-0.009 [-0.030,0.012]	0.012 [-0.005,0.028]	0.017 [-0.006,0.040]	-0.030 [-0.072,0.013]
**Time**	-0.035* [-0.042, -0.028]	-0.002 [-0.005,0.001]	-0.009* [-0.016, -0.002]	-0.005* [-0.010, -0.000]	0.011 [-0.008,0.030]
**Ventilator-Admitted Ratio**	-16.662* [-29.406, -3.918]	0.555 [-10.098,11.208]	-11.074 [-25.332,3.183]	-14.092* [-19.096, -9.089]	-15.681* [-27.872, -3.489]

**Oxygen-Admitted Ratio**	3.734* [0.788,6.680]	4.413* [1.848,6.979]	0.148 [-5.421,5.718]	0.640 [-2.558,3.838]	2.435 [-4.943,9.814]
**LOG (Second doses administered) 14-day delay**	-	-	0.073* [0.006,0.139]	0.104* [0.010,0.198]	-0.375* [-0.668, -0.083]
**Constant**	-1.222 [-4.180,1.736]	-6.707* [-10.447, -2.967]	3.169 [-1.606,7.945]	-3.417 [-8.348,1.514]	-4.846 [-18.956,9.263]
**Observations**	122	128	104	147	83

**Significant at a 95% CI or higher*

Newey West CI in parentheses

The daily new COVID-19 tests and Oxford Health and Containment Index, which records the presence of government NPIs and restrictions, were both found to be a statistically significant determinants of COVID-19 deaths in wave 1. Increasing daily new tests by 1% will reduce the daily deaths by 0.65% (95% CI: 0.26%, 1.04%). While increase in Oxford Health and Containment Index by 1 point will result in 0.03% reduction in daily new deaths (95% CI: -0.05%, -0.005%).

The time trend variable is statistically significant in wave 1, wave 3 and wave 4. The coefficients indicate that, on average, daily COVID-19 deaths decreased at a rate of 0.04% per day (95% CI: -0.04%, -0.03%) as time passed during wave 1. However, daily new deaths reduced at a rate of 0.01% per day during wave 3 (95% CI: -0.02%, 0.002%) and wave 4 (95% CI: -0.01%, 0.00%).

The ventilator to admitted ratio was statistically significant in wave 1, wave 4 and wave 5. The coefficient remains negative throughout these three waves and significant at 95% CI. The coefficients indicates that if ratio of patients on ventilator out of the admitted increases than daily new deaths decrease by 14-17%.

Oxygen to admitted ratio is only significant in wave 1 and 2 at 95% CI, where the coefficient is positive. It means that if the ratio of oxygenated patient out of the total admitted increase than daily new deaths due to COVID-19 will increase by approximately 4%.

Lastly, the number of fully vaccinated people came out to be statistically significant in each of the last three waves. During wave 3, COVID-19 deaths increase by 0.07% (95% CI: 0.006,0.14) as percentage of fully vaccinated people increase by one addition percentage. This rate increased to 0.10% (95% CI: 0.006,0.14) during the wave 4. However, in the fifth wave, daily new deaths due to COVID-19 decrease by 0.38% (95% CI: -0.67, -0.08) as fully vaccinated people increases.

DISCUSSION

Pakistan experienced 5 distinct waves from 3rd April 2020 to 23rd February 2022. Our analysis reflects both the evolution of Pakistan's response, as well as the differential impact of different variants of the virus shaped the contours and features of each wave. Pakistan experienced its initial wave earlier than other South Asian countries including India, Bangladesh, Sri Lanka and Nepal, while peaks for the subsequent waves coincided with those in other countries (31).

The upslope, as seen by the rate of change for testing and cases, was always steeper than during the downward slope of a wave. This pattern follows what is known about infectious epidemics in that cases rise quickly, plateau and then fall, slowly to an endemic state where a low ebb of infections persist in the community in definitely (12). In fact, each wave behaved as a typical epidemic caused by a distinct variant of the virus. Wave 1 was

dominated by B.1 variant, wave 2 by B.1.36 variant, the wave 3 by Alpha/B1.1.7 and Delta/B.1.617.2 variants, wave 4 had majority cases of the Delta/B.1.617.2 variant (28–30) while wave 5 was driven by Omicron/BA.5.2.1.7 (4).

A key challenge faced by Pakistan at the beginning of the pandemic was that there was little prior experience with any pandemic outbreak of such level. Although disease surveillance systems exist, they had not been scaled to manage case surveillance, hospital admissions, daily deaths, and eventually large-scale adult vaccination and event tracking. Pakistan has a federal system of governance where provinces provide health services while the federal ministry provides guidance and coordination. In addition, considerable curative care is in the private sector. To address the potential difficulties in mounting a unified national response to the disease in the face of this diversity, a National Action Plan for COVID-19 was formulated in March 2020 that placed the responsibility for the national response in a National Coordination Committee (NCC) that was headed by the Prime Minister and attended by all federal ministers. The NCC set national policy which was implemented by the National Command and Operation Centre (NCOC) that was co-headed by the military and civilian leadership (32). The NCOC coordinated the management of the extensive lockdowns, other key NPIs such as school closures, limited opening hours for essential businesses (examples of which included grocery stores and pharmacies), closure of borders, cancellation of public events and social gatherings (33,34). This was supported based on an elaborate data gathering and analysis system that that guided daily decisions.

Wave 1 continued the longest and intervals became shorter between each successive wave. Each wave showed unique features, that were determined by the particular variant

that drove that wave, along with the larger context that included the type of the variant driving the wave, the extent and type of preventive interventions and eventually the availability of the vaccine.

Pakistan’s response to COVID-19 evolved over time. For example, Wave 1 had the highest positivity rates and the longest duration, in part due to low initial rates of testing, including very little contact tracing in the early days (35). As testing increased and mobility restrictions tightened, duration of Waves 2 and 3 became shorter. However, by the end of the second wave, intervention fatigue had set in. Implementation was laxer, and these factors contributed to more cases and deaths of any wave during Wave 3. Indeed, the Oxford Health and Containment Index was significant only during Wave 1 in terms of preventing deaths.

In addition to preventive measures, the higher daily COVID-19 cases in waves 3, 4 and 5 may be attributed to highly transmissible Alpha (36,37), Delta (38,39) and Omicron (40) variants, and to easing of severe restrictions such as lockdowns and school closures (41). It is also possible that many cases were missed during Wave 1 due to limited testing. However, the stability of daily testing in waves 3 to 5 suggests a stable equilibrium between the testing system and how cases were being incident – the system was capturing most of the cases from previously recognized populations and locations. It is likely that undiagnosed cases and deaths were few, since as part of the national surveillance, teams kept abreast of burials in large and midsized towns and also periodically canvased opinion of general practitioners about upsurges in respiratory illnesses. On average, Pakistan had fewer cases per million population than neighboring

countries of India, Bangladesh, and Iran, as well as several of the developed countries (31).

As with prevention, clinical management of cases evolved over time. Initially most cases were hospitalized as seen by the high case to hospitalization ratio – only 27% of admissions required oxygen 7% required ventilators during the Wave 1. In fact, there was a correlation between deaths and oxygenation (which was mostly at hospitals) during waves 1 and 2, a pattern that was seen globally. However, with each succeeding wave, use of oxygen increased while ventilators fluctuated within a narrow range, as was also seen in India (52,53). Thus, even as COVID-19 hospitalizations peaked during Wave 3, hospitalization to case ratio increased, and average duration of hospitalization and the use of hospitals for simple oxygenation fell, suggesting hospitals, ICU and ventilators, were increasingly reserved only for the sickest (42). Deaths correlated best with a 21-day delay model rather than a 28-day one, suggesting that most deaths happened early after infection. Higher hospitalizations during the third wave may also have been attributed to the Alpha followed by Delta variants (43–45). By contrast the lower hospitalizations, length of stay and mortality during the fifth wave may be attributed to the Omicron variant that was seen worldwide (46,47), and specifically in South Africa (48) and Brazil (49) during the Omicron waves. Vaccination started halfway during wave 3 and more than half of the eligible population was fully vaccinated (3) by the fifth wave, and may have contributed to lower hospitalizations in Wave 5. Unlike COVID-19 induced major challenges to the healthcare capacity in various countries (50,51), Pakistan was able to build healthcare resources capacity to keep pace with the pandemic. Ventilator and oxygen utilization never exceeded 20% and 24% respectively in wave 3.

Vaccination drive started in Pakistan by the end of February 2021. Despite a slow start, vaccination picked up pace from 26,356 daily vaccinations in Wave 3 to 308,129 in the fourth wave as it was rolled-out to younger population and vaccine supply increased in the country. Average daily deaths did not reduce significantly due to vaccinations during the third and fourth waves (56,57) but showed marked reduction in hospitalizations and deaths towards the end of fourth and during the entire fifth wave (58).

From our regression model, we found that daily new COVID-19 cases were statistically significant determinants of daily new deaths due to COVID-19. The association was also observed from the third wave as both cumulative cases and deaths were the highest, including the average daily deaths which were considerably higher than any other wave, as seen in other countries (54). Secondly, daily new deaths due to COVID-19 increased with patients on oxygenated beds while decreased with patients on ventilators in the initial waves, potentially due to high patients load in hospitals, critical patients were put on oxygen rather than ventilator. Fifth wave experienced the smallest number of daily COVID-19 deaths possibly because it was dominated by the Omicron variant (55).

LIMITATIONS

There are a number of limitations associated with the data used in this paper. While the official data used for the analysis are disaggregated by sub-national level, demographic disaggregation, such as age or gender, are not available. This limits the analysis in terms of the implication of gender and age on COVID-19 deaths. The national data is compiled by aggregating the numbers for each subnational unit in Pakistan. However, such an analysis would be too extensive to depict and therefore our analysis does not account for sub-national differences. It is possible that distinctive cultures, behaviors and differences

in the stringency in enforcement of interventions vary between for regions and may in theory, influence the number of COVID-19 cases and deaths.

Other data sources must be looked like private medical sector, Pharmacological surveillance.

Similarly, data for hospitalizations is also unaccompanied by any information on comorbidities, as this information was not available beyond treating hospitals, losing a level of richness of analysis that includes such comorbidities. Also, data for daily new hospital admissions started becoming available towards the very end of wave 1. Consequently, the average length of hospital stay could not be calculated for this wave.

The official vaccination data available to us at the time of this analysis is not desegregated by the different types of available vaccines, for example Sinopharm, CanSino, Sputnik V and others. Differential impact of each vaccine on COVID-19 deaths in Pakistan would be informative. All of the above limitations notwithstanding, we are confident that this study provides crucial insights into the prevailing trends of COVID-19 in Pakistan in manner that is constructive.

What about of impact this article. Any future practical recommendations must be suggested. Did this article played any role in current situation.

CONCLUSION

Can these findings related and compared with modelling studies conducted in Pakistan

COVID-19 management in Pakistan kept pace with the spread of the disease during five distinct waves. We describe how these waves differed in terms of cases, hospitalizations and deaths and analyze potential reasons for these differences. These lessons, as well as limitations of our approach, provide important lessons for managing future pandemics.

REFERENCES

1. Noreen N, Dil S, Ullah S, Niazi K, Naveed I, Khan NU, et al. Coronavirus disease (COVID-19) Pandemic and Pakistan; Limitations and Gaps. Limitations Gaps Glob Biosecurity [Internet]. 2020;1(3). Available from: https://jglobalbiosecurity.com/articles/63/galley/170/download/

2. Babu GR, Khetrapal S, John DA, Deepa R, Narayan KMV. Pandemic preparedness and response to COVID-19 in South Asian countries. Int J Infect Dis [Internet]. 2021;104:169–74. Available from: https://doi.org/10.1016/j.ijid.2020.12.048

3. NCOC. Vaccine Statistics [Internet]. 2022 [cited 2022 Feb 23]. p. NCOC Official Website. Available from: https://covid.gov.pk/vaccine-details

4. Badar N, Ikram A, Salman M, Umair M, Rehman Z, Ahad A, et al. Genomic characterization of SARS-CoV-2 from Islamabad, Pakistan by Rapid Nanopore sequencing. Available from: https://doi.org/10.1101/2022.02.17.480826

5. Coccia M. The impact of first and second wave of the COVID-19 pandemic in society: comparative analysis to support control measures to cope with negative effects of future infectious diseases. Environ Res [Internet]. 2021;197(April):111099. Available from: https://doi.org/10.1016/j.envres.2021.111099

6. Salyer SJ, Maeda J, Sembuche S, Kebede Y, Tshangela A, Moussif M, et al. The first and second waves of the COVID-19 pandemic in Africa: a cross-sectional study. Lancet [Internet]. 2021;397(10281):1265–75. Available from: http://dx.doi.org/10.1016/S0140-6736(21)00632-2

7. Iftimie S, Lopez-Azcona AF, Vallverdu I, Hernandez-Flix S, De Febrer G, Parra S, et al. First and second waves of coronavirus disease-19: A comparative study in hospitalized patients in Reus, Spain. PLoS One. 2021;16(3 March 2021):1–13.

8. Jalali SF, Ghassemzadeh M, Mouodi S, Javanian M, Kani MA, Ghadimi R, et al. Epidemiologic comparison of the first and second waves of coronavirus disease in Babol, North of Iran. Casp J Intern Med. 2020;11(2):S544–50.

9. Wouters OJ, Shadlen KC, Salcher-Konrad M, Pollard AJ, Larson HJ, Teerawattananon Y, et al. Challenges in ensuring global access to COVID-19 vaccines: production, affordability, allocation, and deployment. Lancet. 2021 Mar 13;397(10278):1023–34.

10. Vanthomme K, Gadeyne S, Lusyne P, Vandenheede H. A population-based study on mortality among Belgian immigrants during the first COVID-19 wave in Belgium. Can demographic and socioeconomic indicators explain differential mortality? SSM - Popul Heal [Internet]. 2021;14:100797. Available from: https://doi.org/10.1016/j.ssmph.2021.100797

11. So MKP, Chu AMY, Tiwari A, Chan JNL. On topological properties of COVID-19: predicting and assessing pandemic risk with network statistics. Sci Rep [Internet]. 2021;11(1):1–14. Available from: https://doi.org/10.1038/s41598-021-84094-z

12. Wasserheit JN, Aral SO. The dynamic topology of sexually transmitted disease epidemics: Implications for prevention strategies. J Infect Dis. 1996;174(SUPPL. 2).

13. Sibiri H, Prah D, Zankawah SM. Containing the impact of COVID-19: Review of

Ghana's response approach. Heal Policy Technol [Internet]. 2021;10(1):13–5. Available from: https://doi.org/10.1016/j.hlpt.2020.10.015

14. Olufadewa II, Adesina MA, Ekpo MD, Akinloye SJ, Iyanda TO, Nwachukwu P, et al. Lessons from the coronavirus disease 2019 (COVID-19) pandemic response in China, Italy, and the U.S.: a guide for Africa and low- and middle-income countries. Glob Heal J [Internet]. 2021;5(1):56–61. Available from: https://doi.org/10.1016/j.glohj.2021.02.003

15. He D, Zhao S, Xu X, Lin Q, Zhuang Z, Cao P, et al. Low dispersion in the infectiousness of COVID-19 cases implies difficulty in control. BMC Public Health. 2020;20(1):2–5.

16. Van Nguyen Q, Cao DA, Nghiem SH. Spread of COVID-19 and policy responses in Vietnam: An overview. Int J Infect Dis [Internet]. 2021;103:157–61. Available from: https://doi.org/10.1016/j.ijid.2020.11.154

17. Sharma A, Borah SB, Moses AC. Responses to COVID-19: The role of governance, healthcare infrastructure, and learning from past pandemics. J Bus Res [Internet]. 2021;122(102):597–607. Available from: https://doi.org/10.1016/j.jbusres.2020.09.011

18. Seong H, Hyun HJ, Yun JG, Noh JY, Cheong HJ, Kim WJ, et al. Comparison of the second and third waves of the COVID-19 pandemic in South Korea: Importance of early public health intervention. Int J Infect Dis [Internet]. 2021;104:742–5. Available from: https://doi.org/10.1016/j.ijid.2021.02.004

19. OxCGRT. OxCGRT COVID Policy Tracker Data [Internet]. 2021. Available from: https://github.com/OxCGRT/covid-policy-tracker/tree/master/data/timeseries

20. Hale T, Anania J, Angrist N, Boby T, Cameron-Blake E, Folco M Di, et al. Variation in government responses to COVID-19, Version 12.0 [Internet]. BSG Working Paper Series. 2021. Available from: https://covidtracker.bsg.ox.ac.uk/stringency-scatter

21. Baud D, Qi X, Nielsen-Saines K, Musso D, Pomar L, Favre G. Real estimates of mortality following COVID-19 infection. Lancet Infect Dis [Internet]. 2020;20(7):773. Available from: http://dx.doi.org/10.1016/S1473-3099(20)30195-X

22. Elezkurtaj S, Greuel S, Ihlow J, Michaelis EG, Bischoff P, Kunze CA, et al. Causes of death and comorbidities in hospitalized patients with COVID-19. Sci Rep [Internet]. 2021;11(1):1–9. Available from: https://doi.org/10.1038/s41598-021-82862-5

23. Marschner IC. Estimating age-specific COVID-19 fatality risk and time to death by comparing population diagnosis and death patterns: Australian data. BMC Med Res Methodol [Internet]. 2021;21(1):1–10. Available from: https://doi.org/10.1186/s12874-021-01314-w

24. Asirvatham ES, Sarman CJ, Saravanamurthy SP, Mahalingam P, Maduraipandian S, Lakshmanan J. Who is dying from COVID-19 and when? An

Analysis of fatalities in Tamil Nadu, India. Clin Epidemiol Glob Heal [Internet]. 2021;9:275–9. Available from: https://www.sciencedirect.com/science/article/pii/S2213398420302189

25. Borobia AM, Carcas AJ, Pérez-Olmeda M, Castaño L, Bertran MJ, García-Pérez J, et al. Immunogenicity and reactogenicity of BNT162b2 booster in ChAdOx1-S-primed participants (CombiVacS): a multicentre, open-label, randomised, controlled, phase 2 trial. Lancet. 2021;398(10295):121–30.

26. Li J, Hou L, Guo X, Jin P, Wu S, Zhu J, et al. Heterologous prime-boost immunization with CoronaVac and Convidecia. medRxiv [Internet]. 2021;(20):2021.09.03.21263062. Available from: http://medrxiv.org/content/early/2021/09/06/2021.09.03.21263062.abstract

27. Liu X, Shaw RH, Stuart AS, Greenland M, Dinesh T, Provstgaard-Morys S, et al. Safety and Immunogenicity Report from the Com-COV Study – a Single-Blind Randomised Non-Inferiority Trial Comparing Heterologous And Homologous Prime-Boost Schedules with An Adenoviral Vectored and mRNA COVID-19 Vaccine. SSRN Electronic Journal. 2021.

28. Umair M, Ikram A, Rehman Z, Haider A, Badar N, Ammar M, et al. Genomic diversity of SARS-CoV-2 in Pakistan during fourth wave of pandemic. medRxiv [Internet]. 2021;92(51):2021.09.30.21264343. Available from: http://medrxiv.org/content/early/2021/10/03/2021.09.30.21264343.abstract

29. Basheer A, Zahoor I. Genomic epidemiology of SARS-CoV-2 divulge B.1, B.1.36, and B.1.1.7 as the most dominant lineages in first, second, and third wave of SARS-CoV-2 infections in Pakistan. Microorganisms [Internet]. 2021 [cited 2022 Sep 21];9(12). Available from: https://doi.org/10.3390/microorganisms9122609

30. Shakeel M, Irfan M, Nisa Z un, Farooq S, Ain N ul, Iqbal W, et al. Genome sequencing and analysis of genomic diversity in the locally transmitted SARS-CoV-2 in Pakistan. Transbound Emerg Dis [Internet]. 2022; Available from: http://tree.bio.ed.ac.uk/software/Fig.tree/

31. Ritchie H, Mathieu E, Rodés-Guirao L, Appel C, Giattino C, Ortiz-Ospina E, et al. Coronavirus Pandemic (COVID-19) [Internet]. OurWorldInData.org. 2020. Available from: https://ourworldindata.org/coronavirus

32. National Institute of Health Pakistan. National Action Plan for Corona virus disease (COVID-19) Pakistan [Internet]. 2020. Available from: https://www.nih.org.pk/wp-content/uploads/2020/03/COVID-19-NAP-V2-13-March-2020.pdf

33. Gul A. Pakistan Seals Borders, Shuts Schools, Bans Public Events Over Coronavirus. Voanews.com [Internet]. 2020 Mar 13;COVID-19 Pandemic. Available from: https://www.voanews.com/a/science-health_coronavirus-outbreak_pakistan-seals-borders-shuts-schools-bans-public-events-over/6185785.html

34. Chandir S, Siddiqi DA, Setayesh H, Khan AJ. Impact of COVID-19 lockdown on routine immunisation in Karachi, Pakistan. Lancet Glob Heal [Internet]. 2020;8(9):e1118–20. Available from: https://www.thelancet.com/journals/langlo/article/PIIS2214-109X(20)30290-4/fulltext

35. Habib M, Abbas M. Facing the Threat of COVID-19 in Pakistan: A Nation's Dilemma. Value Heal Reg issues. 2021 May;24:90–5.

36. Volz E, Mishra S, Chand M, Barrett JC, Johnson R, Geidelberg L, et al. Assessing transmissibility of SARS-CoV-2 lineage B.1.1.7 in England The COVID-19 Genomics UK (COG-UK) consortium. Nature [Internet]. 2021;593. Available from: https://doi.org/10.1038/s41586-021-03470-x

37. Davies NG, Abbott S, Barnard RC, Jarvis CI, Kucharski AJ, Munday JD, et al. Estimated transmissibility and impact of SARS-CoV-2 lineage B.1.1.7 in England. Science (80-) [Internet]. 2021;372(6538). Available from: http://science.sciencemag.org/

38. Zhan Y, Yin H, Yin J-Y. (Delta) Variant of SARS-CoV-2: features, transmission and potential strategies. Int J Biol Sci [Internet]. 2022;2022(5):1844–51. Available from: https://www.ijbs.com//creativecommons.org/licenses/by/4.0/

39. Kumar S, Tripathi V, Zhang J, Hu Z, Huang X, Fu S, et al. Differences in Clinical Characteristics Between Delta Variant and Wild-Type SARS-CoV-2 Infected Patients. Front Med | www.frontiersin.org [Internet]. 2019;1:792135. Available from: www.frontiersin.org

40. Tian D, Sun Y, Xu H, Ye Q. The emergence and epidemic characteristics of the highly mutated SARS-CoV-2 Omicron variant. 2022; Available from: https://www.gisaid.org/

41. Provinces announce easing lockdown even as Pakistan witnesses record rise in coronavirus cases [Internet]. DAWN. 2020 [cited 2022 Aug 1]. Available from: https://www.dawn.com/news/1555575

42. Karagiannidis C, Windisch W, McAuley DF, Welte T, Busse R. Major differences in ICU admissions during the first and second COVID-19 wave in Germany [Internet]. Vol. 9, The Lancet Respiratory Medicine. 2021 [cited 2022 Oct 7]. p. e47–8. Available from: https://doi.

43. Twohig KA, Nyberg T, Zaidi A, Thelwall S, Sinnathamby MA, Aliabadi S, et al. Hospital admission and emergency care attendance risk for SARS-CoV-2 delta (B.1.617.2) compared with alpha (B.1.1.7) variants of concern: a cohort study. Lancet Infect Dis [Internet]. 2021; Available from: https://www.thelancet.com/journals/laninf/article/PIIS1473-3099(21)00475-8/fulltext

44. Shiehzadegan S, Alaghemand N, Fox M, Venketaraman V. Analysis of the Delta Variant B.1.617.2 COVID-19. Clin Pr [Internet]. 2021;11:778–84. Available from: https://doi.org/10.3390/clinpract11040093

45. Sheikh A, McMenamin J, Taylor B, Robertson C. SARS-CoV-2 Delta VOC in Scotland: demographics, risk of hospital admission, and vaccine effectiveness. Lancet [Internet]. 2021 Jun 26 [cited 2022 Oct 7];397(10293):2461–2. Available from: http://www.thelancet.com/article/S0140673621013581/fulltext

46. Chenchula S, Karunakaran P, Sharma S, Chavan M. Current evidence on efficacy of COVID-19 booster dose vaccination against the Omicron variant: A systematic review. J Med Virol [Internet]. 2022 Jul 1 [cited 2022 Oct 7];94(7):2969–76. Available from: https://pubmed.ncbi.nlm.nih.gov/35246846/

47. Ulloa AC, Buchan SA, Daneman N, Brown KA. Estimates of SARS-CoV-2 Omicron Variant Severity in Ontario, Canada. JAMA [Internet]. 2022 Apr 5 [cited 2022 Oct 7];327(13):1286–8. Available from: https://jamanetwork.com/journals/jama/fullarticle/2789408

48. Maslo C, Friedland R, Toubkin M, Laubscher A, Akaloo T, Kama B. Characteristics and Outcomes of Hospitalized Patients in South Africa during the COVID-19 Omicron wave Compared with Previous waves. JAMA - J Am Med Assoc. 2022 Feb 8;327(6):583–4.

49. Martins-Filho PR, de Souza Araújo AA, Quintans-Júnior LJ, Soares BDS, Barboza W de S, Cavalcante TF, et al. Dynamics of hospitalizations and in-hospital deaths from COVID-19 in northeast Brazil: a retrospective analysis based on the circulation of SARS-CoV-2 variants and vaccination coverage. Epidemiol Health [Internet]. 2022 [cited 2022 Oct 7];44:e2022036. Available from: https://pubmed.ncbi.nlm.nih.gov/35413166/

50. Mateen BA, Wilde H, Dennis JM, Duncan A, Thomas N, McGovern A, et al. Hospital bed capacity and usage across secondary healthcare providers in England during the first wave of the COVID-19 pandemic: a descriptive analysis. BMJ Open [Internet]. 2021 Jan 26 [cited 2022 Oct 7];11(1). Available from: https://pubmed.ncbi.nlm.nih.gov/33500288/

51. Mahendradhata Y, Andayani NLPE, Hasri ET, Arifi MD, Siahaan RGM, Solikha DA, et al. The Capacity of the Indonesian Healthcare System to Respond to COVID-19. Front Public Heal. 2021 Jul 7;9:887.

52. Kumar G, Mukherjee A, Sharma RK, Menon GR, Sahu D, Wig N, et al. Clinical profile of hospitalized COVID-19 patients in first & second wave of the pandemic: Insights from an Indian registry based observational study. Indian J Med Res [Internet]. 2021 May 1 [cited 2022 Oct 7];153(5–6):619. Available from: /pmc/articles/PMC8555588/

53. Budhiraja S, Indrayan A, Aggarwal M, Jha V, Jain D, Tarai B, et al. Differentials in the characteristics of COVID-19 cases in wave-1 and wave-2 admitted to a network of hospitals in North India [Preprint]. medRxiv [Internet]. 2021 Jun 27 [cited 2022 Oct 7];2021.06.24.21259438. Available from: https://www.medrxiv.org/content/10.1101/2021.06.24.21259438v1

54. Anscombe C, Lissauer S, Thole H, Rylance J, Dula D, Menyere M, et al. A

comparison of four epidemic waves of COVID-19 in Malawi; an observational cohort study [Preprint]. medRxiv [Internet]. 2022 [cited 2022 Oct 7]; Available from: /pmc/articles/PMC9298135/

55. Abdullah F, Myers J, Basu D, Tintinger G, Ueckermann V, Mathebula M, et al. Decreased severity of disease during the first global omicron variant covid-19 outbreak in a large hospital in tshwane, south africa. Int J Infect Dis [Internet]. 2022 Mar 1 [cited 2022 Oct 7];116:38–42. Available from: https://pubmed.ncbi.nlm.nih.gov/34971823/

56. Jabłońska K, Aballéa S, Toumi M. The real-life impact of vaccination on COVID-19 mortality in Europe and Israel. Public Health. 2021 Sep 1;198(May):230–7.

57. Lopez Bernal J, Andrews N, Gower C, Gallagher E, Simmons R, Thelwall S, et al. Effectiveness of Covid-19 Vaccines against the B.1.617.2 (Delta) Variant. N Engl J Med [Internet]. 2021 Aug 12 [cited 2022 Oct 7];385(7):585–94. Available from: https://pubmed.ncbi.nlm.nih.gov/34289274/

58. Xavier CR, Oliveira RS, Vieira V da F, Lobosco M, Dos Santos RW. Characterisation of Omicron Variant during COVID-19 Pandemic and the Impact of Vaccination, Transmission Rate, Mortality, and Reinfection in South Africa, Germany, and Brazil. BioTech [Internet]. 2022 Jun 1 [cited 2022 Oct 7];11(2). Available from: /pmc/articles/PMC9264399/

List of Legends

Table 1. Augmented Dicky-Fuller tests for unit roots

Table 2. Descriptive characteristics of each COVID-19 wave

Table 3. Ordinary least squares (OLS) regression results for COVID-19 deaths

Figure 1. COVID-19 waves and daily new cases

S1 Table. Calculated ratio variables and their descriptions

Figure

Click here to access/download;Figure;Fig1.tif

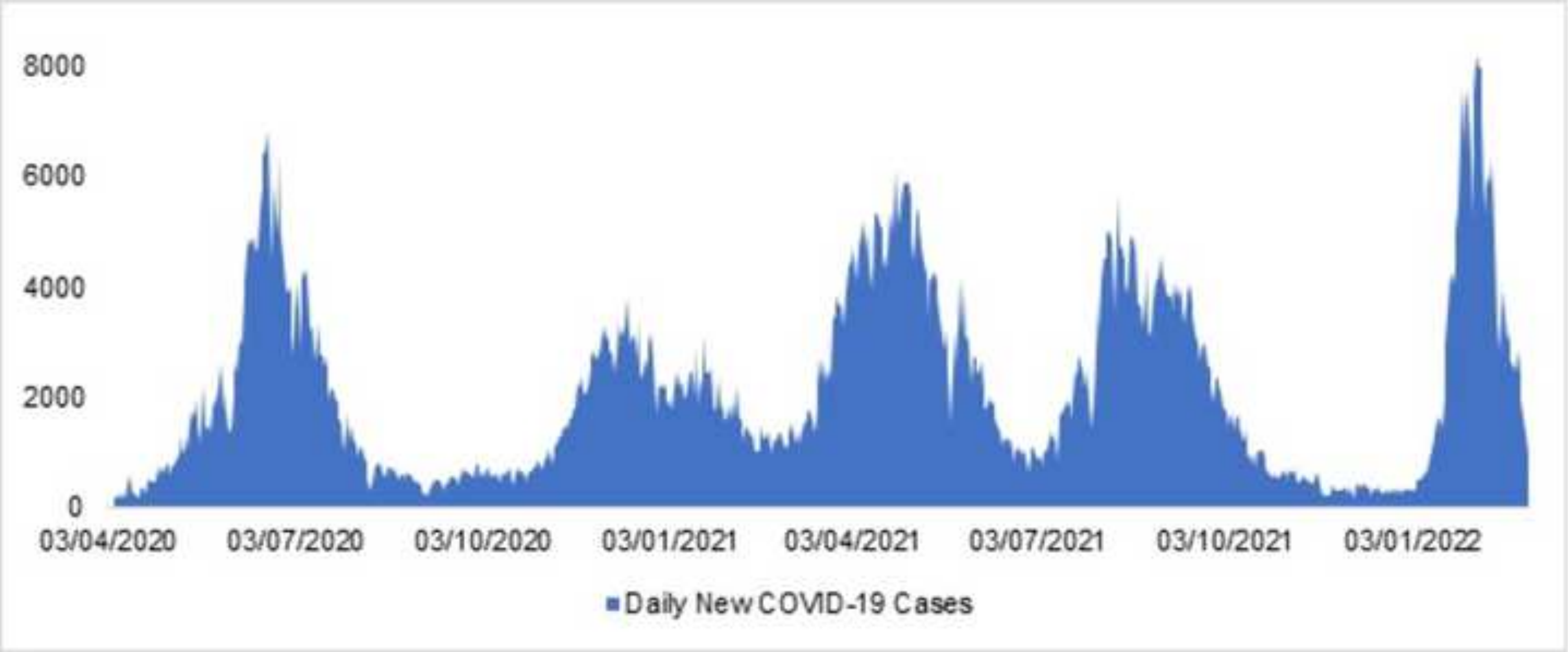

Click here to access/download

Supporting Information - Compressed/ZIP File Archive

Supporting information.zip
