# Supplementary material for: COVID-19 in Pakistan: A national analysis of five pandemic waves
Source: PLoS One. 2023 Dec 29;18(12):e0281326. doi: 10.1371/journal.pone.0281326 (PMC10756537; doi:10.1371/journal.pone.0281326)
Supplement: Attachment — Submitted filename: Reviewer Report.docx [file pone.0281326.s003.docx]

**Reviewer Report**

Dear Huzaifa Ahmad Cheema,

Thank you for the invitation to revise and resubmit the manuscript titled: “COVID-19 in Pakistan: A national analysis of five pandemic waves”. We also thank the reviewers for their time and feedback on this manuscript. Their collective comments have prompted us to improve the manuscript substantially. Below we present a point-by-point response to the comments made.

| **Comment** | **Response** |
| --- | --- |
| **Reviewer #1:** |  |
| The manuscript is a nation-wide review of the first five waves of COVID-19 in Pakistan. Although much has been written about COVID-19, nation-wide data from an under-resourced setting are lacking, thus this manuscript is a valuable additional to the medical literature. It offers a fairly comprehensive review of the epidemiology of these waves in Pakistan, covering a number of important and interesting variables. The manuscript is well written, completed an appropriate analysis, and is well-cited. Although I rarely make this determination in reviewing manuscripts for the first time, I believe it is ready to accept for publication as it currently stands. My congratulations to the authors on their excellent work. | We are very grateful for your review and comments on our manuscript. We are happy to contribute to the COVID-19 literature, especially for a country like Pakistan. |
| **Reviewer #2:** |  |
| The main observation concerns the objectives of this analysis: they should be clearly and unambiguously stated. The conclusions should state whether (and how) they were met. | We have improved the objective of the manuscript in the last paragraph of introduction. Lines 80-87  We have also updated the conclusion to state how the objectives were met. Lines 374-390 |
| Page 6, line 128: the manuscript comprises statistical analysis and inferences. | We have improved the wording. Line 132 -133 |
| Page 5 and page 7 -- lists of themes and independent variables in the regression model, respectively: the presumed connection between the two lists needs to be revised and clarified; if there is no connection, the lists should nevertheless be revised for more clarity and meaningfulness (namely, remove all ambiguity). | We have made some changes in the data and variables section to distinguish between the themes and independent variables in the regression model. |
| Page 8, line 162: Statistical(!) tests were performed to insure that the required assumptions for the regression model were met. | We have replaced the wording of this line as suggested. Line 166-169 |
| Page 5, line 93: double-check the reference to Table 1. | The reference is updated to table 2 which presents the length of each wave in days with their respective dates. Line 97 |
| Page 6, Model specification: the model itself should be independent of the statistical package employed to analyze the data; I would kindly suggest specifying the statistical package at the end of the section. | We have moved the statistical package information to the end of the section. Line 172-173. |
| Page 6, line 132 (equation): t must be detailed/explained; equation should also have a number. | We have assigned equation number and explanation of t is provided. Line 128-145 |
| Pages 13-14, Table 3: the outcome/dependent variable(s) must be clearly stated, together with the number of data records; a landscape layout should be considered, as well. I also recommend having a more informative caption: the explicit/unambiguous connection with the regression model. | We have clearly stated the dependent variable, added data records in the headings and also updated the caption for more clarity. Line 230-231 |
| Page 3 – Introduction: the citations and references' numbering is incorrect; please revise. | We have revised and corrected the references in our entire manuscript. |
| Table S1 – length of hospital stay: the terms "hospital admissions" and "new hospital admissions “are confusing. "Hospital admissions" seems to refer to the number of hospitalized patients at a given time. Please revise the definition and remove any ambiguity. | We have improved the wording of the definition to make it more clear. |
| Page 3, line 51: please revise the SARS-CoV-2 strains, namely the Alpha strain notation) and also revise the tables that include the notation. | We have revised the SARS-COV-2 strains name both in line 54-55 and table 2. |
| Page 8, Table 3: the connection with the regression model's predictors should be explicit (for example by adding an additional column). | Along with specifying the dependent variable in Table 3, we have included variable names as previously specified in equation 1 in Model Specification section to improve clarification. |
| Pages 9-12, Table 2: the table is very long and confusing due to the lack of horizontal lines. Authors might consider splitting into meaningful separate tables with landscape layout. | We have added lines to separate each of the broad themes of indicators, shortened variable names, and revised for consistency to improve readability. Now Table 2 is set on a single page. |
| Authors should use a consistent notation of the pandemic waves throughout the manuscript (for  example, choose one of "wave 3" or "third wave"). | We have made the notation of the pandemic waves consistent throughout the manuscript now. |
| Please double-check all specifications of confidence intervals throughout the manuscript (an example of faulty notation is on page 13, line 225). | We have checked all the specification of confidence interval throughout the manuscript. We have removed the percentage sign from the confidence intervals. |
| **Reviewer #3:** |  |
| describe little bit about specific criteria in abstract method | We have described the criteria for a wave in the methods section in abstract |
| The rationale and justification for this article must be incorporated | We have made changes to the last paragraph of the introduction section to state our rationale and justification for this article clearly. |
| Please mention impact of study | Impact of the study is elaborated in the conclusion section of this manuscript. |
| Provincial specific cultural and social, religious, education status, social mobilization, public compliance, administrative, infrastructural (etc) factors must be considered for NPI. Did these factors may have played a role as confounder. | The manuscript is primarily based on the national rather than provincial level. In addition, uniform NPIs were enforced across the country regardless of the province like school closure to reduce the spread of COVID-19 in the general population. |
| **Reviewer #4:** |  |
| Regarding the statement in the summary, "A one percent increase in vaccinations increased daily new COVID-19 deaths by 0.10% (95% CI: 0.01, 0.20)," it is essential to clarify the source of this data, since it is not clear in the text or in the tables. The current phrasing might inadvertently imply a causal relationship between vaccination and an increase in daily deaths. It is crucial to revise this sentence to avoid the misunderstanding that vaccination directly led to higher mortality. Although the data allows for different interpretations, a clear causal link cannot be established solely from these numbers. | We have specified the source of data in the methods section of the abstract. We have also changed the wording of the vaccination variable interpretation. We have specifically used the term association now so readers do not consider it as a causal relationship. |
| Consider incorporating a comparative analysis between the official death data examined in your study and the excess mortality data. This comparison is pertinent since the cumulative death count over the five waves appears to be considerably lower than the excess mortality figures reported in early 2022 (https://doi.org/10.1016/S0140-6736(21)02796-3). Additionally, the insights provided in this article could benefit from a cross-reference to related research, as indicated by this source: https://doi.org/10.1038/s41586-022-05522-2. | The excess mortality figures are derived from statistical modeling, and they estimate the total number of deaths in Pakistan, including those that may not have been officially reported. However, our data source for Pakistan is based solely on the figures reported by the Government of Pakistan. Comparing these reported statistics with the estimated total deaths due to COVID-19 may not yield meaningful or logically consistent results, as we would be essentially comparing confirmed cases with a broader estimate that includes unreported cases. This is, however, an interesting comparison which we may consider for our future research. |
| Enhance the clarity of the visual representation by marking the initiation and conclusion of each wave within figure 1. To provide a more comprehensive perspective, it would be valuable to disaggregate the data by gender, including both cases and fatalities. Furthermore, it could be beneficial to highlight the time points at which vaccination initiatives were introduced. | Unfortunately, we have national aggregated data so we cannot separate it by gender, age etc. However, we’ve included daily new deaths due to COVID-19 and marked start and end of waves, as well as the starting date of vaccination campaign. |
| Concerning Table 3, the significance of the asterisk accompanying all values of LOG (Daily NewCOVID-19 Cases) – 21-day delay) requires clarification. Please elucidate the purpose of these asterisks to aid the reader's understanding of the presented data. | The asterisks represents the level of significance at 95% or more confidence level. This is already reported under the table 3. |
